# Slowly Progressive Melanoma Differentiation-Associated Gene 5 (MDA-5) Interstitial Lung Disease Following COVID-19 Infections: A Pulmonary Puzzle

**DOI:** 10.7759/cureus.81584

**Published:** 2025-04-01

**Authors:** Sara A Armstrong, Denise Sese, Aravind Menon

**Affiliations:** 1 Internal Medicine, Medical University of South Carolina, Charleston, USA; 2 Pulmonary and Critical Care Medicine, Medical University of South Carolina, Charleston, USA

**Keywords:** amyopathic dermatomyositis, autoimmunity, covid-19, immunosuppression, interstitial lung disease, mda-5

## Abstract

Melanoma differentiation-associated gene 5 (MDA-5) autoimmunity has been increasingly recognized in association with interstitial lung disease (ILD), particularly in the context of viral infections. While MDA-5 ILD is typically rapidly progressive with high mortality, emerging evidence suggests that COVID-19 may induce a distinct, more indolent phenotype.

We report two cases of MDA-5 ILD following COVID-19 infection with a slowly progressive course.

In Case 1, an 87-year-old woman with a history of breast cancer, chronic obstructive pulmonary disease (COPD), and a prior COVID-19 infection developed progressive dyspnea. Imaging revealed new-onset ILD with subpleural reticulations and traction bronchiectasis. A myositis antibody panel was positive for MDA-5 autoantibodies. She was diagnosed with MDA-5 ILD and initiated on corticosteroids, later transitioned to mycophenolate. She has since remained clinically stable over the past two years.

In Case 2, an 84-year-old man with a history of smoking and asbestos exposure developed severe dyspnea and hypoxia after a mild COVID-19 infection. CT imaging revealed diffuse ground-glass opacities and interlobular septal thickening. He was diagnosed with MDA-5 ILD and Group 1 and 3 pulmonary hypertension. He was treated with mycophenolate and pulmonary vasodilators, leading to symptom stabilization.

These cases illustrate a possible post-COVID-19 endotype of MDA-5 ILD that follows a slower disease trajectory, distinct from the classical rapidly progressive phenotype. The association between viral infections and MDA-5 autoimmunity is supported by prior studies demonstrating viral-induced interferon signaling and MDA-5 gene activation. Given the indolent nature of disease progression in our patients, a tailored immunosuppressive approach was pursued, resulting in the stabilization of symptoms and pulmonary function.

These cases contribute to the growing recognition of post-COVID-19 MDA-5 ILD as a unique clinical entity. Further research is needed to elucidate the precise mechanisms underlying virus-induced autoimmunity and to guide treatment strategies for this emerging ILD subtype.

## Introduction

Melanoma differentiation-associated gene 5 (MDA-5) antibodies are strongly associated with clinically amyopathic dermatomyositis (CADM), a rare autoimmune condition that often manifests with skin findings seen in dermatomyositis (DM) but without skeletal muscle involvement. In some cases, DM may be entirely absent or may present later in the disease course, making the diagnosis more challenging [1]. A complication of CADM is interstitial lung disease (ILD) [2], with MDA-5-positive ILD most commonly presenting as a rapidly progressive form often carrying a high risk of morbidity and mortality within months of onset [3]. Patients usually experience progressive dyspnea, cough, and hypoxemia with high-resolution CT imaging revealing ground-glass opacities, interlobular septal thickening, and signs of fibrosis [3]. However, emerging evidence suggests that MDA-5 autoimmunity may present along a broader clinical spectrum, including more indolent, slowly progressive forms of ILD. This is particularly relevant in the post-COVID-19 era, as viral infections may act as potential triggers for autoimmunity [4]. MDA-5, a ribonucleic acid (RNA) sensor and key pattern recognition receptor for viruses like SARS-CoV-2, may play a role in a shared pathogenic pathway between COVID-19 and MDA-5-associated ILD [4]. Here, we present two cases of slowly progressive MDA-5-positive ILD following COVID-19 infection, highlighting the need for individualized disease monitoring and tailored treatment strategies. 

## Case presentation

Case 1

An 87-year-old woman presented to the pulmonary clinic with a year-long history of shortness of breath. Her past medical history included breast cancer status post-lumpectomy, chronic obstructive pulmonary disease (COPD), distant history of secondary spontaneous pneumothorax from emphysema requiring talc pleurodesis, and a mild COVID-19 infection one year prior to presentation. She was not hospitalized, nor did she require supplemental oxygen. Her symptoms began shortly after the resolution of her COVID-19 infection. She reported worsening dyspnea with an inability to perform certain household tasks. She was a former smoker with a 30-pack-year history and quit four years ago. She did not have notable environmental or occupational exposures. She denied fever, chills, dysphagia, angina, rashes, myalgias, weakness, or Raynaud's. She did not have weight gain or lower extremity edema. The review of systems was only notable for dry eyes and mouth.

She was normotensive, in sinus rhythm, and breathing appropriately on room air. The physical exam was notable for bilateral crackles with decreased air entry bilaterally. She had normal muscle strength.

Her comprehensive metabolic panel (CMP), complete blood count (CBC), erythrocyte sedimentation rate (ESR), C-reactive protein (CRP), creatine kinase (CK), cyclic citrullinated peptide (CCP), and aldolase were unremarkable. Rheumatoid factor (RF) was 72.1 (<30). A myositis antibody panel returned positive for MDA-5 antibodies at 32 (normal: <20). Spirometry showed moderate obstruction with restriction. She had a forced expiratory volume in one second/forced vital capacity (FEV1/FVC) of 66, FVC 1.88 L (77% predicted), FEV1 1.23 L (67% predicted), and a severely reduced diffusion capacity (DLCO) of 32% predicted.

**Figure 1:**
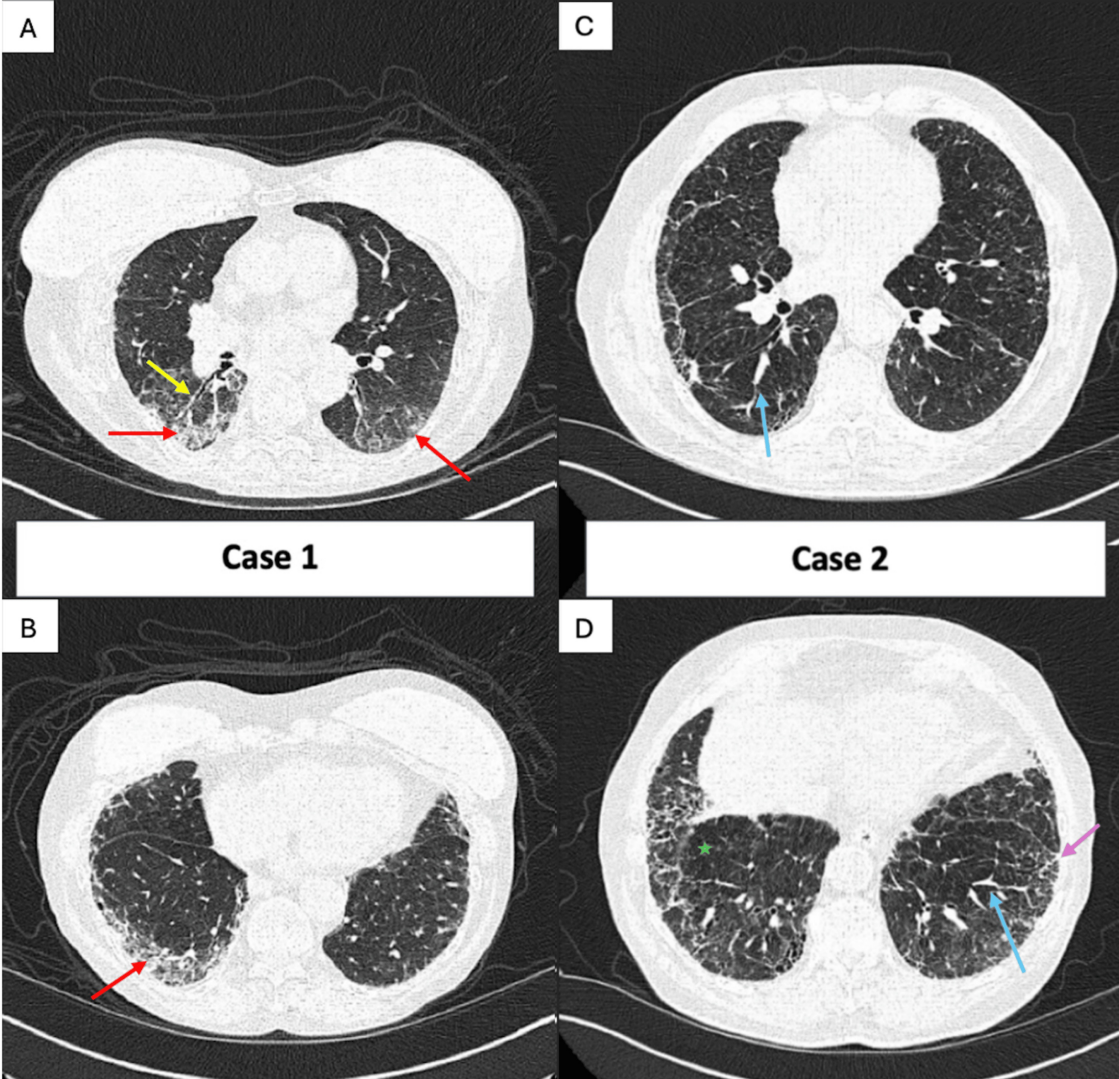
High-resolution helical CT scans. The studies image the lung apices through the bases without IV contrast material in both inspiratory and expiratory phases (A) Yellow arrow highlighting traction bronchiectasis and bronchiolectasis; red arrow indicating ground-glass opacities. (B) Red arrow illustrating lower lobe-predominant ground-glass opacities. (C) Blue arrow illustrating interlobular septal thickening. (D) Blue arrow again illustrating interlobular septal thickening; green star marking an area of mosaic attenuation with abnormal air trapping; light purple arrow indicating subpleural reticulations.

CT of the chest showed bilateral upper lobe-predominant areas of destructive centrilobular emphysema with bilateral lower lobe-predominant ground-glass opacities with subpleural interstitial infiltrates (Figure 1A, 1B). There was early evidence of architectural distortion with traction bronchiectasis and bronchiolectasis (Figure 1A). No honeycombing was noted. Prior CT imaging from two years prior showed emphysematous changes but was otherwise without pre-existing ILD. A transthoracic echocardiogram (TTE) showed left ventricular ejection fraction (LVEF) of 65-70%, impaired left ventricular relaxation, and mild tricuspid regurgitation, with an estimated pulmonary artery (PA) pressure of 40-45 mmHg.

Case 2

An 84-year-old man presented to the pulmonary clinic with progressive dyspnea. His symptoms began following a three-day hospitalization for a mild COVID-19 infection, not requiring high flow or ICU admission. He was discharged with supplemental oxygen of up to 5 liters/minute and developed chronic but stable dyspnea. He was active prior to his infection with no symptoms of dyspnea and cough and no functional limitations. At presentation, he was unable to walk more than 15 feet without dyspnea. He reported ambulatory desaturations up to 70-80%. He denied fever, chills, dysphagia, angina, rashes, myalgias, and Raynaud's. He had no weight gain or lower extremity edema. The review of systems was notable for dry eyes and mouth. 

He had a 90-pack-year smoking history and quit 35 years ago. He worked in the Navy as a welder with exposures to asbestos and fiberglass installation. He denied any family history of lung cancer or pulmonary fibrosis, but his sister was diagnosed with scleroderma in her 40s.

He was mildly hypertensive and in normal sinus rhythm with a respiratory rate of 16 breaths/minute. The physical examination was notable for bilateral rhonchi in the lower lobes and facial telangiectasia. He did not have muscle or joint tenderness.

His CMP, CBC, RF, CCP, CK, extractable nuclear antigen (ENA) panel, and aldolase were unremarkable. His anti-nuclear antigen was positive with a speckled pattern, 1:1280 titer. A myositis antibody panel returned positive for MDA-5 antibodies at 27 (normal: <20).

CT imaging illustrated diffuse ground-glass opacities, subpleural reticulations, and bilateral effusions with notable mosaic attenuation and interlobular septal thickening (Figure 1C, 1D). Spirometry showed mild obstructive disease with the following results: FEV1/FVC of 67, FEV1 2.79 L (113 %predicted), and FVC 4.19 L (112% predicted). The DLCO, not corrected for hemoglobin, was substantially reduced (18% predicted). TTE showed LVEF 55-60% with severe pulmonary hypertension and right ventricular systolic pressure (RVSP) estimated at 86 mmHg. A TTE obtained prior to his COVID-19 infection did not show right ventricular abnormalities. He underwent a right heart catheterization with the following pressure readings: right atrium 8 mmHg, right ventricle 104/10 mmHg, PA 95/32 (55) mmHg, and pulmonary capillary wedge pressure 12 mmHg. The calculated pulmonary vascular resistance (PVR) was 10.5 Wood units (WU) (normal: <2 WU). After inhaled nitric oxide (iNO), his PA pressure decreased to 71/30 (43) mmHg, and his PVR decreased to 7.1 WU. Although there was a decrease in his right-sided filling pressures after iNO, the mean PA pressure remained above 40 mmHg with a mild decrease in cardiac output. Given this non-response with findings of pre-capillary pulmonary hypertension and a probable Group 3 component, he was started on tadalafil and inhaled treprostinil.

## Discussion

Both cases demonstrate CT changes such as traction bronchiectasis and bronchiolectasis, reticular infiltrates, interlobular septal thickening, and ground-glass opacities (Figure 1A-1D). A myositis antibody panel was obtained for each patient which returned positive for MDA-5 autoantibodies. An infectious workup, including blood and sputum cultures, a respiratory viral panel, and *Pneumocystis jirovecii* testing, was unremarkable. Connective tissue-related disease, anti-neutrophil cytoplasmic autoantibody (ANCA)-associated vasculitis, anti-synthetase syndrome, Sjögren's disease, systemic sclerosis, and drug-induced ILD were additionally ruled out based on autoimmune panels and exposure history. Both patients were diagnosed with MDA-5 ILD following a COVID-19 infection. Notably, they did not exhibit the typical rapidly progressive ILD course often associated with MDA-5 pulmonary involvement. They also did not exhibit the typical cutaneous manifestations, such as ulcerations, mechanic's hands, Gottron's sign, or a heliotrope rash. The first patient showcased a lack of ILD findings on imaging prior to her COVID-19 infection. The second patient, although without prior imaging to show a lack of ILD, presented with dyspnea shortly after a COVID-19 infection. He notably had facial telangiectasias shortly after as well. Prior to the COVID-19 infection, his previous TTE did not show signs of RV dysfunction. He was additionally in his usual state of health and was able to walk one hour a day regularly prior to infection.

Each patient saw pulmonary and rheumatology teams for multidisciplinary management. 

**Figure 2:**
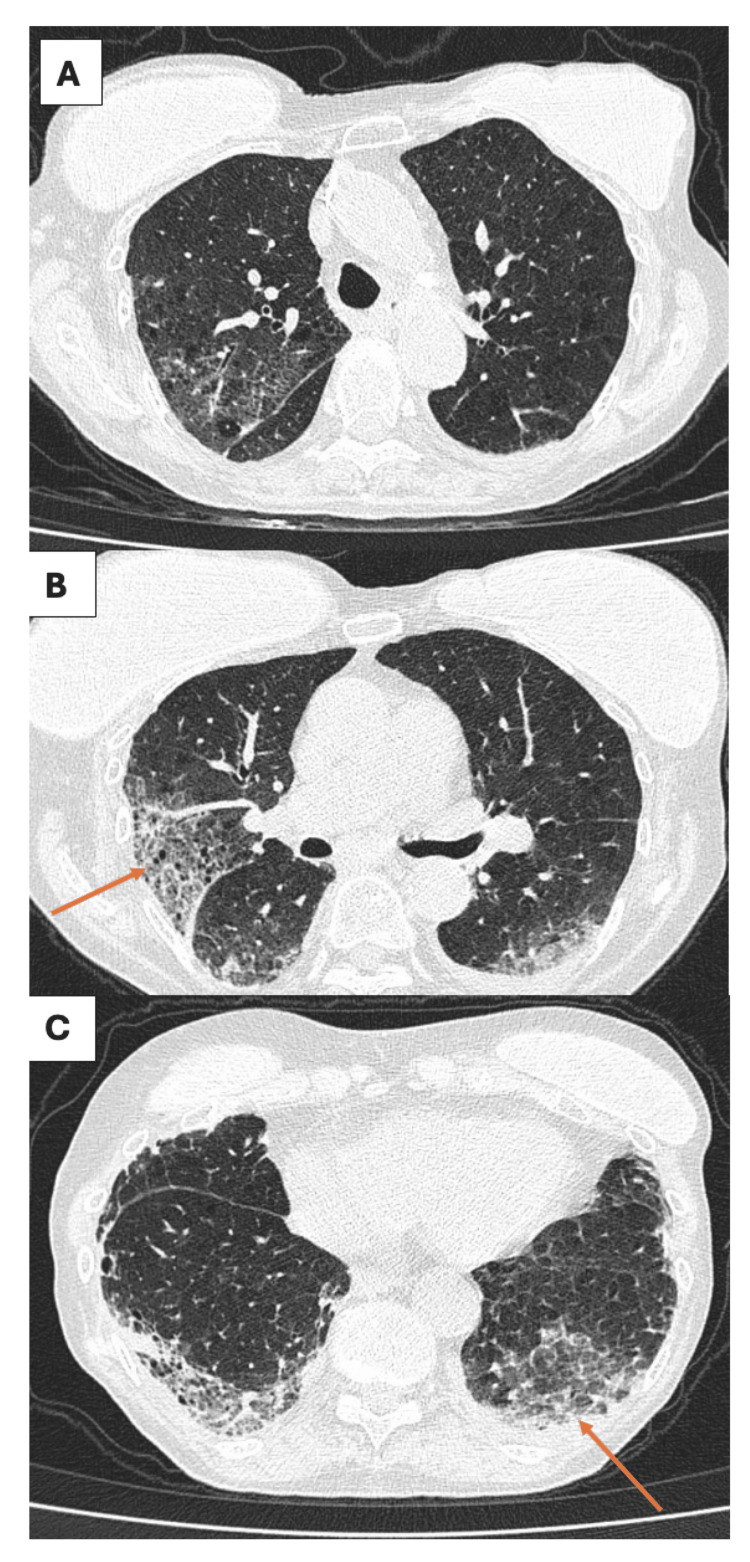
High-resolution follow-up CT imaging: Case 1 (B, C) Orange arrows depicting the progression in the lower lobe-predominant ground-glass opacities.

Our first patient initially began steroid therapy while undergoing an autoimmune workup. Immune modulation was initially avoided due to a traumatic wound on her lower extremity, which, despite the absence of active infection, required negative pressure wound therapy. On interval follow up, she was noted to have stability in her clinical symptoms and lung function. Consequently, her steroids were tapered, and she was eventually started on mycophenolate 1000 mg twice daily. Mycophenolate was chosen not only for its steroid-sparing properties but also for its favorable side effect profile. After three months, she was diagnosed with squamous cell carcinoma of her lower extremity, prompting mycophenolate discontinuation. Following the removal of the carcinoma, mycophenolate was restarted while acknowledging the increased risk of immunosuppression and recurrent malignancy. Eight months after the diagnosis of MDA-5 ILD, repeat spirometry (Table 1) showed an FEV1/FVC of 67, FVC 1.97 L (83% predicted), FEV1 1.31 L (74% predicted), and a reduced diffusion capacity of 41% predicted (increased from 32% previously). Follow-up imaging after one year showed mild progression in her ILD (Figure 2A-2C). Her initial and one-year follow-up six-minute walk tests showed a total distance reduction of 320 feet (Table 2).

**Table 1:** One-year progression in PFTs: Case 1 The FEV1 indicates how much air a patient can forcefully exhale in one second. The FVC is the total amount of air a patient can forcefully exhale after taking a deep breath. The FEV1/FVC ratio helps assess lung function and diagnose obstructive or restrictive diseases. The DLCO assesses the efficiency of gas exchange in the lungs. There is some improvement in the above PFTs after treatment was initiated, however still illustrating a mixed obstructive/restrictive pattern with an improvement from severely reduced DLCO to only moderately reduced. PFTs: pulmonary function tests; FEV1: forced expiratory volume in one second; FVC: forced vital capacity; FEV1/FVC: ratio of FEV1 and FVC; DLCO: diffusing capacity of the lung for carbon monoxide

PFT values	Initial	Follow-up
FEV1	1.23 L (67% predicted)	1.31 L (74% predicted)
FVC	1.88 L (77% predicted)	1.79 L (83% predicted)
FEV1/FVC	66	67
DLCO	32% predicted	41% predicted

**Table 2:** Initial and one-year follow-up six-minute walk tests: Case 1 The SAO2 and total distance walked during six minutes is a standardized measurement to assess functional exercise capacity. As illustrated in the above table, the distance our patient from Case 1 walked decreased by 320 feet over a year once treatment was initiated. However, the SAO2 during ambulation remained the same on room air. SAO2: oxygen saturation of the arterial blood

Six-minute walk test	Initial	Follow-up
Distance	1020 ft	700 ft
Lowest SAO2 (room air)	90%	90%

**Figure 3:**
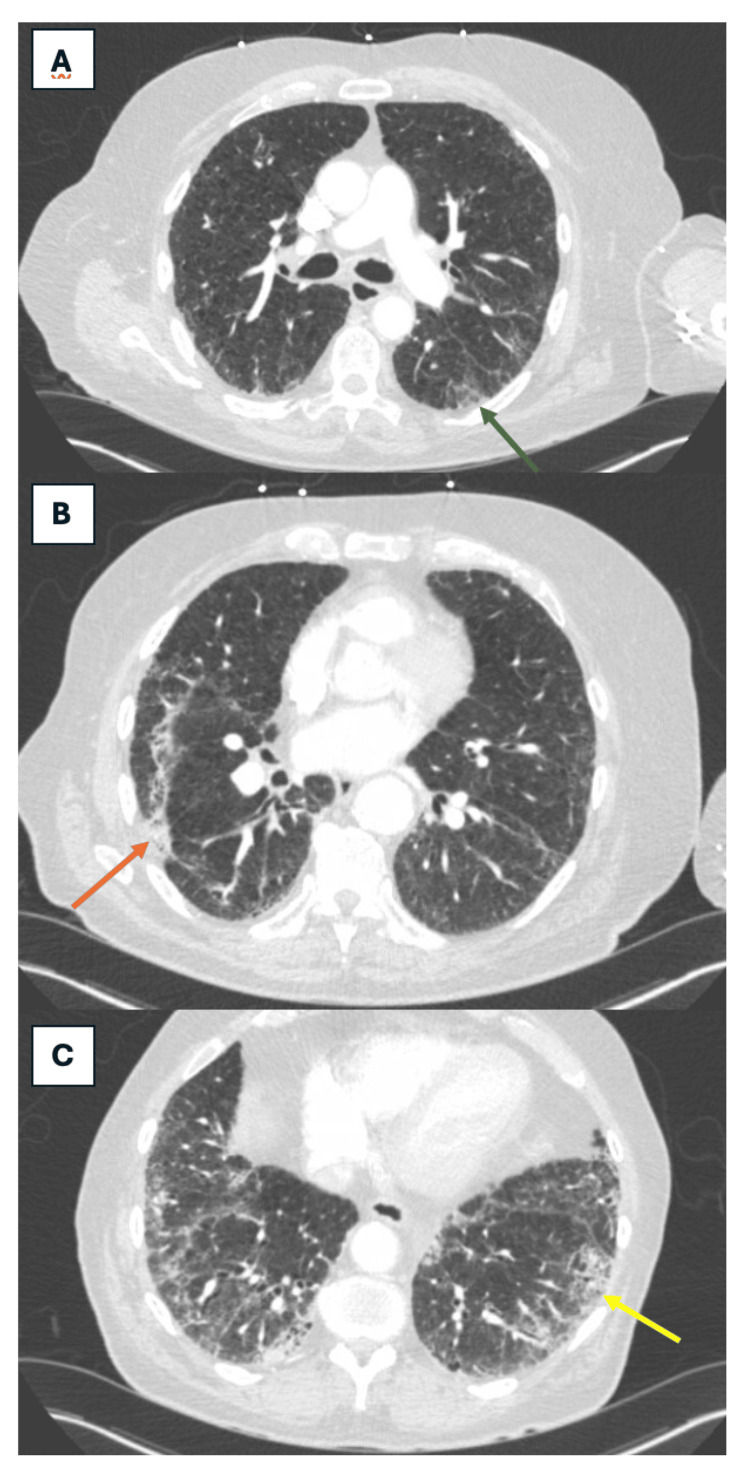
One-year follow-up CT imaging: Case 2 (A) Dark green arrow depicting new ground-glass opacities. (B) Orange arrow pointing to the progression of reticular opacities and fibrosis, along with ground-glass opacities. (C) Yellow arrow showcasing subpleural reticulations as well as septal thickening.

Our second patient was initiated on mycophenolate. He experienced significant gastrointestinal side effects leading to Myfortic® initiation (mycophenolate sodium formulation) which he tolerated at a dose of 720 mg twice daily. Throughout this period, his pulmonary function tests (Table 3) remained stable (FEV1/FVC 67, 90% predicted; DLCO 19% predicted), and follow-up imaging showed mild progression in his ILD (Figure 3A-3C). His initial and one-year follow-up six-minute walk tests were without much change (Table 4). Given his stability, he did not warrant additional antifibrotic therapy. He was referred to pulmonary rehabilitation and palliative care for symptom management and required 6 L oxygen at rest compared to 4 L when he initially presented. Monitoring has included pulmonary function tests every six months. For his Group 1 and 3 pulmonary hypertension, a repeat TTE showed a decreased RVSP of 54 mmHg following the initiation of tadalafil and treprostinil. His ventilation/perfusion scan was without defects. It was ultimately concluded that the Group 1 component of his pulmonary hypertension was secondary to both MDA-5 ILD and a prior COVID-19 infection. Studies have shown that persistent endothelial-mesenchymal transition (EndMT), mediated by MDA-5 proteins, can contribute to pulmonary vascular remodeling [5]. We suspect that the ILD component of his disease, along with progressive lung tissue thickening and scarring, leading to restricted blood flow and increased pulmonary artery pressure, played a role in the development of both the type 3 and type 1 components.

**Table 3:** One-year progression in PFTs: Case 2 The FEV1 indicates how much air a patient can forcefully exhale in one second. The FVC is the total amount of air a patient can forcefully exhale after taking a deep breath. The FEV1/FVC ratio helps assess lung function and diagnose obstructive or restrictive diseases. The DLCO assesses the efficiency of gas exchange in the lungs. In the above table, there is minimal change in the above values. These PFTs exhibit obstruction with a severely reduced DLCO. PFTs: pulmonary function tests; FEV1: forced expiratory volume in one second; FVC: forced vital capacity; FEV1/FVC: ratio of FEV1 and FVC; DLCO: diffusing capacity of the lung for carbon monoxide

PFT values	Initial	Follow-up
FEV1	2.79 L (113% predicted)	2.56 (100% predicted)
FVC	4.19 L (112% predicted)	3.71 (106% predicted)
FEV1/FVC	67	67
DLCO	18% predicted	19% predicted

**Table 4:** Initial and one-year follow-up six-minute walk tests: Case 2 The SAO2 and total distance walked during six minutes is a standardized measurement to assess functional exercise capacity. As illustrated in the above table, the patient from Case 2 walked 50 additional feet after one year of treatment. However, his SAO2 decreased to 89% with ambulation. SAO2: oxygen saturation of the arterial blood

Six-minute walk test	Initial	Follow-up
Distance	650 ft	700 ft
Lowest SAO2 (5 L nasal cannula)	96%	89%

It has been proposed that the production of MDA-5 autoantibodies is triggered by viral infections, including COVID-19 [6]. Several helicase proteins, such as retinoic-acid-inducible protein I (RIG-I) and MDA-5 (also known as IFIH1), are implicated in viral double-stranded RNA recognition [7]. Although COVID-19 is a single-stranded RNA virus (ssRNA), recent in vivo and in vitro studies have shown that COVID-19 can induce the production of the MDA-5 gene in the lungs, potentially leading to chronic lung pathology such as ILD [4,8-10].

MDA-5 is implicated in several disease states, most notably DM, and is highly associated with rapidly progressive ILD, a condition with high mortality. RP-ILD is the major cause of death in patients with MDA-5 DM [3,11]. According to several cohort studies, over 80% of patients with RP-ILD die within the first six months [8]. Anti-MDA-5 antibody-positive patients can develop ILD without DM or amyopathic DM. In fact, the amyopathic subtype of MDA-5 appears to be more frequent in patients with positive MDA-5 antibodies [12]. However, the majority of patients (60-70%) exhibit the classical skin manifestations [12].

The interest in MDA-5 autoimmunity increased significantly during the COVID-19 pandemic with the identification of a new endotype of MDA-5 autoimmunity that behaved differently [4]. Unlike the more commonly known rapidly progressive ILD, a group of patients manifested a milder, chronic, insidious course. A study of 21 patients diagnosed with anti-MDA-5 DM highlighted the clinical spectrum of the disease, revealing that six patients had asymptomatic ILD, while seven presented with chronic, symptomatic ILD [1,13].

Studies have also observed an increase in the overall MDA-5 positivity rate when comparing the post-COVID-19 to pre-COVID-19 time frame [4], which we believe further solidifies an association between MDA-5 and COVID-19. Although further investigation is needed to understand the precise mechanisms and patient susceptibility to post-COVID-19 autoimmunity, there may be a slowly progressive MDA-5 ILD associated with COVID-19. This newly identified endotype may warrant alternative immunosuppressive therapies and could be associated with an improved prognosis. In line with this evolving understanding, recent guidelines now recommend upfront combination therapy with dual or triple immunosuppression for patients with systemic autoimmune rheumatic disease-associated interstitial lung disease (SARD-ILD), particularly for rapidly progressive disease [2,14]. Specifically, for individuals with confirmed or suspected MDA-5-related ILD, early initiation of aggressive immunosuppression is favored over monotherapy to mitigate disease progression.

## Conclusions

Previous cohort studies have demonstrated the association between viral cytokine induction and MDA-5 gene transcription, revealing a new subtype of MDA-5 ILD. These two cases may also illustrate a potential shared pathogenic pathway between COVID-19 and MDA-5 antibodies. Further research is needed to understand how different viruses can induce interferons and MDA-5 gene transcription. The COVID-19 pandemic has brought forth an increased number of cases encompassing virus-associated autoimmunity. Multi-center efforts across nations are essential to recognize the underlying burden of these cases.

These two cases highlight a subset of MDA-5 ILD that follows a more indolent and slowly progressive course, in contrast to the traditionally aggressive and high-mortality presentation of the disease. While these findings offer insight into potential variability in disease progression, there are notable limitations to consider. As an observational case series, the generalizability of these cases is inherently limited. Additionally, while a possible link between COVID-19 and MDA-5 autoimmunity is suggested, a definitive causal relationship cannot be established based on case reports alone. Further research is needed to better understand the full spectrum of MDA-5 ILD and its potential triggers.

This milder disease trajectory may influence further management and prognosis. There are no clear recommendations for the management of MDA-5 ILD; however, immunosuppressive therapy remains a mainstay treatment option. Our patients responded well and were well-controlled on mycophenolate/Myfortic®. Each patient, however, deserves an individualized approach to immunosuppressant treatment to optimize outcomes. Other treatment options include glucocorticoids (e.g., prednisone), calcineurin inhibitors (e.g., tacrolimus, cyclosporine), cyclophosphamide, rituximab, and Janus kinase (JAK) inhibitors (e.g., tofacitinib, baricitinib), often tailored based on disease severity, progression, as well as side effect profile.
